# Pluripotency of induced pluripotent stem cells

**DOI:** 10.1186/2049-1891-3-5

**Published:** 2012-02-28

**Authors:** Lan Kang, Shaorong Gao

**Affiliations:** 1 National Institute of Biological Sciences, NIBS, Beijing, 102206, China

**Keywords:** induced pluripotent stem cells, pluripotency, reprogramming, tetraploid complementation

## Abstract

Recent studies have demonstrated that differentiated somatic cells from various mammalian species can be reprogrammed into induced pluripotent stem (iPS) cells by the ectopic expression of four transcription factors that are highly expressed in embryonic stem (ES) cells. The generation of patient-specific iPS cells directly from somatic cells without using oocytes or embryos holds great promise for curing numerous diseases that are currently unresponsive to traditional clinical approaches. However, some recent studies have argued that various iPS cell lines may still retain certain epigenetic memories that are inherited from the somatic cells. Such observations have raised concerns regarding the safety and efficacy of using iPS cell derivatives for clinical applications. Recently, our study demonstrated full pluripotency of mouse iPS cells by tetraploid complementation, indicating that it is possible to obtain fully reprogrammed iPS cells directly from differentiated somatic cells. Therefore, we propose in this review that further comprehensive studies of both mouse and human iPS cells are required so that additional information will be available for evaluating the quality of human iPS cells.

## Introduction

An iPS cell is induced from a non-pluripotent cell, but possesses pluripotency similar to that of ES cells. Takahashi and Yamanaka (2006) first achieved this landmark breakthrough by reprograming mouse embryonic fibroblasts (MEFs) into this new type of pluripotent stem cell via the ectopic expression of only four transcription factors, namely Oct4, Sox2, Klf4 and c-Myc. This new procedure circumvented the need for an oocyte, which is required by an earlier method of generating customized pluripotent stem cells termed somatic cell nuclear transfer (SCNT)-mediated nuclear reprogramming [1-3]. Since the discovery of iPS cells, the field has attracted a great amount of scientific and public attention because of the undefined mechanism by which the developmental potential of the cells is reverted and the potential for clinical applications using patient specific iPS cells. The generation of iPS cells from individual patients has raised the hope of treatments for numerous degenerative and genetic diseases [4-11].

Unlike normal fertilization or the generation of SCNT-ES cells, the creation of iPS cells is a longer process that results in a heterogeneous mixture of cells with various developmental potentials. In the primary culture, iPS cells are usually present together with the original somatic cells, transformed cells and partially reprogrammed cells. Indeed, iPS cells are only approximately 0.1% to 1% of the total cells used for reprogramming. Moreover, only very small proportions of these cells are fully reprogrammed based on stringent criteria for evaluating pluripotency. Therefore, it is necessary to establish a molecular standard to distinguish fully reprogrammed iPS cells from those that are partially reprogrammed, especially for human iPS cells that may eventually be used for clinical applications.

In the present review, we will summarize the most recent progress toward understanding the pluripotency of mouse iPS cells at the functional and molecular levels. We anticipate that further studies will be undertaken to improve our understanding of the determination and regulation of pluripotency in human iPS cells.

### Differentiation Potential of Stem Cells

The capacity for differentiation into other cell types under the appropriate conditions is the most important property of early embryonic cells and stem cells. Based on distinct differentiation capabilities, stem cells can be subdivided into pluripotent, multipotent and unipotent stem cells [12]. Only zygotes and the blastomeres of early embryos (before the 8-cell stage in mice) during development possess totipotency. Totipotent embryos differentiate into more than 200 types of cells that belong to the three germ layers of development, as well as extraembryonic tissues *in vivo*, thereby producing new life. After this stage, blastomeres lose totipotency and undergo the first cell fate determination. At the blastocyst stage, a small number of blastomeres develop into the pluripotent inner cell mass (ICM) and the rest differentiate into trophectoderm that forms the extraembryonic tissues and supports embryonic development. The ICM differentiates further into the three germ layers (ectoderm, mesoderm and endoderm) and then into the entire body. This type of developmental capacity is termed pluripotency. Importantly, the ICM can be isolated and maintained *in vitro *to derive ES cells, which can be maintained in the same pluripotent state as the ICM [13,14]. Numerous tissue-specific stem cells, including hematopoietic stem cells, are multipotent and can differentiate into various cell types within the same cell lineage. The spermatogonial stem cell (SSC) is one example of a unipotent tissue-specific stem cell, because SSCs can only differentiate into spermatozoa.

The differentiation potential of stem cells is confirmed by various differentiation assays. Multipotent and unipotent stem cells should have the ability to differentiate into specific cell types after transplantation under the appropriate *in vivo *conditions or by *in vitro *culture with the appropriate stimuli. However, it is not practical to differentiate pluripotent stem cells into all of the possible cell types of an organism *in vitro*, although an embryoid body (EB) that forms the three germ layers can be induced. Subcutaneous transplantation of pluripotent stem cells into an immune deficient mouse produces a teratoma. The formation of teratomas has been used as the most preliminary assay for testing the pluripotency of mouse pluripotent stem cells *in vivo*. A more stringent assay for testing pluripotency is to generate chimeric mice with a germ line transmission ability. However, the chimera assay may not convincingly represent the full pluripotency of pluripotent stem cells.

**Figure 1:**
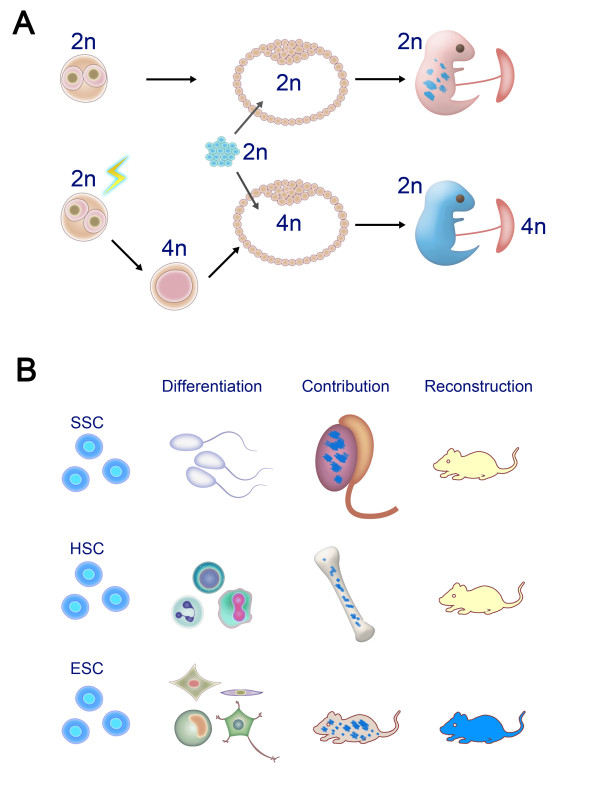
**Developmental potential of induced pluripotent stem (iPS) cells**. A). Chimera and tetraploid embryo complementation assays for evaluating the pluripotency of pluripotent stem cells. Chimeric mice are produced by aggregation of iPS cells with normal diploid embryos. For tetraploid embryo complementation, the full-term organism is produced by iPS cells, whereas the extraembryonic placenta is derived from a tetraploid embryo that is generated by electric fusion of a 2-cell stage embryo. B). Functional assays for evaluating the differentiation potential of stem cells. Spermatogonial stem cells are unipotent stem cells and can differentiate into sperm as well as contribute toward formation of the testes after transplantation. Hematopoietic stem cells are multipotent stem cells and can differentiate into various hematopoietic cells and contribute toward bone marrow. Moreover, hematopoietic stem cells can reconstruct the entire hematopoietic system of irradiated mice. ES cells are pluripotent stem cells and can theoretically differentiate into all cell types of an organism. Following transplantation into normal diploid blastocysts, ES cells contribute toward the formation of all tissues of the chimera. Furthermore, ES cells can reconstruct the entire organism following a tetraploid embryo complementation assay.

Tetraploid blastocyst complementation remains the most stringent assay for testing the pluripotency of pluripotent stem cells. Tetraploid blastocysts are produced via the fusion of 2-cell stage embryos and are developmentally defective by only forming extraembryonic tissues *in vivo *[15]. Interestingly, this developmental characteristic of tetraploid embryos is exactly the opposite of pluripotent stem cells. As expected, ES cells with full pluripotency compensate for the developmental deficiency of tetraploid embryos, and a full-term organism can be produced from pluripotent stem cells together with extraembryonic tissues derived from tetraploid embryos (Figure 1A) [16,17]. In this manner, ES cells differentiate into all of the various types of fetal cells, tissues and organs, which organize into the organism and truly demonstrate the pluripotency of ES cells. A tetraploid complementation assay may also be considered a type of reconstruction assay similar to the multipotency test for hematopoietic stem cells, but the assay reconstructs the whole fetus instead of only the hematopoietic system (Figure 1B). This information is more useful than *in vitro *differentiation of stem cells because it can clearly demonstrates that stem cells possess greater potential for differentiation compared that of other stem cell types.

### Functional Evaluation of Mouse iPS Cell Pluripotency

The landmark achievement by Takahashi and Yamanaka revealed that ectopic expression of four transcription factors in differentiated MEFs can induce nuclear reprogramming to form iPS cells with a typical ES cell morphology. These iPS cells express pluripotency genes and produce teratomas following subcutaneous transplantation into immune deficient nude mice. However, live chimeric mice could not be produced by implanting the original iPS cells into normal fertilized embryos [18]. Therefore, the original iPS cells were not fully pluripotent iPS cells. Subsequently, adult chimeras with germ line transmission ability were generated from iPS cells with improved quality [19-21]. However, it remains uncertain whether fully pluripotent iPS cells can be induced because full-term animals could not be produced from iPS cells via tetraploid complementation, even with extensive efforts [21].

To demonstrate fully pluripotent iPS cells, numerous iPS cell lines underwent tetraploid complementation assays, and live pups were finally generated in three independent laboratories including our own [22-24]. After these studies were performed, the data collectively demonstrated that iPS cells are functionally comparable with that of ES cells. Follow-up experiments provided further evidence that iPS cells derived from fetal somatic cells are not the only type of inducible pluripotent cells. Indeed, iPS cells derived from adult somatic cells can also be fully pluripotent [25]. Furthermore, our recent studies have shown that iPS cells that are reprogrammed with only three factors (without c-Myc) can be fully pluripotent, because viable mice can be produced entirely from the iPS cells [26]. However, it should be noted that the success of producing mice consisting of only iPS cells was likely due to the large number of iPS cell lines being examined, and the majority of iPS cell lines that were tested did not produce viable mice.

### Differences between Fully Pluripotent iPS Cells and Non-fully Pluripotent iPS Cells

To further characterize the differences between fully pluripotent iPS cell lines and non-fully pluripotent iPS cell lines, gene expression was compared between a variety of ES cells and iPS cell lines. A small number of transcripts encoded within the imprinted Dlk1-Dio3 gene cluster on chromosome 12qF1, particularly Glt2 and Rian, are aberrantly silenced in most iPS cell lines that poorly contribute toward chimeras and fail to support the development of iPS cell-derived organisms using tetraploid embryo complementation [25,27]. In contrast, fully pluripotent iPS cell lines exhibit normal expression levels of these genes in this region. Subsequently, the gene expression status of this region has been proposed as a candidate marker for evaluating the quality of iPS cell lines.

**Figure 2:**
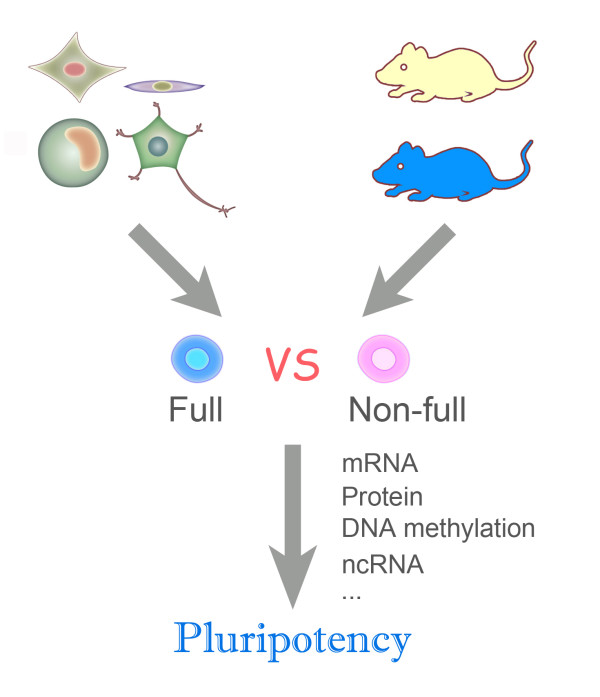
**Molecular analyses of iPS cells**. High throughput analyses of mRNA, protein, DNA methylation and non-coding RNA of fully pluripotent and non-fully pluripotent mouse iPS cells would assist our understanding of the pluripotency regulation of iPS cells. iPS cell lines derived from the same somatic cell, but with varying differentiation potentials should be used, and a sample pool consisting of various iPS cell groups derived from multiple cell types should be used to reach a consensus on iPS cell pluripotency.

However, silencing of the Dlk1-Dio3 gene cluster does not appear to be the only underlying cause of incomplete pluripotency. Although the pluripotency of most iPS cell lines that were tested correlated well with the expression status of these genes, some exceptions existed, in particular Oct4, Sox2 and Klf4 used to derive iPS cell lines [26]. Moreover, the fact that Gtl2 knock-out mice are viable challenges the importance of Gtl2 in determining the pluripotency of iPS cells [28]. It has been reported that treatment by a histone deacetylase inhibitor reactivates the silenced Dlk1-Dio3 cluster in partially reprogrammed iPS cells and rescues the ability of iPS cells to support full-term development of iPS cell-derived mice [25]. However, this study remains debatable because the effects of the inhibitor are very complicated. Therefore, we propose that the quality of iPS cells may not be determined by only one gene cluster, and that further comprehensive studies are necessary for discovering additional candidate genes that may synergistically contribute toward the quality of iPS cells. We suggest that a comprehensive comparison of DNA methylation, gene expression and non-coding RNAs using mouse iPS cell lines derived from the same somatic cells with varying developmental potentials would provide a greater understanding of the regulation of pluripotency. Additionally, a sample pool consisting of various of iPS cell lines derived from multiple cell types and derivation strategies with varying genetic backgrounds should be analyzed to reach a consensus (Figure 2).

### Molecular Comparison of Human iPS Cells versus ES Cells

Unlike mouse iPS cells, in which pluripotency is tested by tetraploid complementation assay, evaluating the pluripotency of human iPS cells and ES cells is considered much more preliminary. Currently, the most stringent assay for testing the pluripotency of human pluripotent stem cells is teratoma formation, which only evaluates the differentiation potential to form the three germ layers or a small number of specific cell types, but can not indicate full pluripotency. Recently, molecular analyses of human ES cells and iPS cells using genome wide high-throughput assays have allowed more quantitative comparisons to be performed.

By comparing gene expression profiles, studies from several independent groups have suggested that human iPS cells generally resemble human ES cells, but some recurrent differential gene expression signatures have been observed [29-32]. These differences indicate both insufficient silencing of donor cell-specific genes and insufficient induction of ES cell-specific gene expression. Furthermore, this original memory and incomplete reprogramming appear to attenuate upon extended culture because late passage human iPS cells possess a gene expression profile that is more similar to human ES cells. However, these differential gene expression profiles are considered to be stochastic and are affected by the *in vitro *micro-environment. Upon the sample pool being augmented, the two pluripotent cell types are not consistently distinguished [33,34].

In addition to the transcriptional profiles, the differential methylation of specific CpG islands is suggested to be distinguishable between human iPS cells and ES cells [35]. Variations in CpG methylation, histone modifications and incomplete reprogramming of non-CpG methylation regions that are proximal to centromeres and telomeres in human iPS cells have been observed in genome-wide profiles of DNA methylation at a single-base resolution [36]. However, Guenther et al. (2010) [33] confirmed that there is little difference between ES cells and iPS cells with respect to genome-wide maps of nucleosomes with histone H3K4 and H3K27 trimethylation, which supports the previous study of H3K4 and H3K27 trimethylation levels in promoter regions [29].

In our opinion, there are several possibilities regarding the cause of these controversies from studies that use similar technologies. First, several studies have suggested that passaging of iPS cells plays an important role in determining the properties of iPS cells [29,37,38]. This is understandable because early-passage iPS cell lines may not have completed the reprogramming process. It will be interesting to investigate whether this is true for all iPS cell lines and if so, evaluation of the reprogramming process should be extended to late passages, and the characterization and application of iPS cells should also be standardized with this aspect.

Second, analytical methods and data interpretation greatly influence study conclusions. For example, the threshold used for the calculation of statistical significance can result in biases. Therefore, controversies based on similar datasets suggest that any potential differences between iPS cells and ES cells are very small. Thus far, no specific significant group has been found.

Third, the properties of various iPS cell and ES cell lines are stochastic. Varying conclusions can be drawn because different cell lines, culture conditions and manipulations are used, and fluctuations can occur within identical microenvironments. Therefore, we suggest increasing the number of tested iPS cell lines to reduce the variation caused by the inadequate comparisons between iPS cell lines.

### Treating iPS Cells as ES Cells for Application

Although the derivation of iPS cells and ES cells is markedly different, there are significant similarities between them. However, a complication that researchers encounter in the application of iPS cells is the heterogenous mixture of cells with various developmental potentials, regardless of which derivation strategy is used. Irrespective of the similarity between fully reprogrammed iPS cells and ES cells, incompletely reprogrammed iPS cells remain present. Therefore, a major consideration for clinical application of iPS cells in the future is to set the minimum criteria to exclude low quality iPS cell lines.

In our opinion, it is not necessary to document the similarity between human iPS cells and ES cells. Human ES cells are also artificial products, and a gold standard is not available for the evaluation of ES cell quality. Clinically, normal cells that can efficiently differentiate into particular cell types are required for regenerative medicine. The use of transdifferentiation for direct conversion of somatic cells into other types of somatic cells has been proposed as an alternative strategy to obtain functional cells for therapy [39-44]. However, the efficacy of the resultant cells would need to be confirmed that the shortened telomeres in aged cells are elongated in the converted cells and if not, the usefulness of these cells for cell-based therapy would be unclear. Thus, iPS cells remain the most attractive cell source for clinical purposes.

We propose that the minimum requirements for the clinical use of human iPS cells are: 1) a normal karyotype, because some sub-karyotypic alterations are observed during reprogramming and a normal karyotype is an important factor for ES cell characterization; 2) a normal genotype. Human iPS cell derivation strategies should be improved to preserve the integrity of donor cells, and the method applied without transgenes to include mRNA and protein-mediated induction assisted by small molecules [45,46]; 3) the activation of pluripotency networks. This would ensure that iPS cells possess the necessary self-renewal ability and differentiation potential; and 4) a specific differentiation ability. This is the most important criteria and represents the usefulness of iPS cells for specific cell-based therapies.

The epigenetic memory from the original donor cells in incompletely reprogrammed iPS cells would affect the differentiation tendency of early-passage iPS cells [37,38]. As previously discussed, iPS cell lines that are stably maintained to late passages should be used. A small number of iPS cell lines have exhibited reduced differentiation efficiencies toward a particular cell type [47], and cell-line-specific differences in DNA methylation and gene expression profiles could lead to an *in vitro *differentiation propensity [48]. Then, primary exclusion may have a positive effect by increasing the differentiation efficiency, but more convenient examination standards should be applied.

## Conclusion

The direct reprogramming of differentiated somatic cells into iPS cells has enabled us to investigate the molecular events during cell fate choice, as well as the potential clinical use of iPS cells to cure numerous diseases. We have demonstrated the full pluripotency of mouse iPS cells in a functional manner via tetraploid complementation. However, molecular standards for distinguishing iPS cells with various pluripotencies remain to be established. Thus, further comprehensive studies are required for improving our understanding of the regulation of pluripotency within mouse iPS cells. More importantly, similar studies to compare human iPS cells will provide information to establish minimal molecular criteria for evaluating the quality of human iPS cells in the future.

## Competing interests

The authors declare that they have no competing interests.

## Authors' contributions

LK and SG wrote and approved the final manuscript.
